# Assessment of Fruit Traits and Antioxidant Capacity in Wild and Cultivated Genotypes of *Ziziphus* sp.

**DOI:** 10.3390/plants14010134

**Published:** 2025-01-05

**Authors:** Radu Liviu Șumălan, Dana Maria Copolovici, Manuela Crișan, Florin Stănică, Renata Maria Șumălan, Andreea Lupitu, Simona Ioana Vicas, Silvia Mot, Lucian Copolovici, Sorin Ciulca

**Affiliations:** 1 Faculty of Engineering and Applied Technologies, University of Life Sciences “King Mihai I” from Timisoara, 119 Calea Aradului, 300645 Timisoara, Romania; radusumalan@usvt.ro (R.L.Ș.); renatasumalan@usvt.ro (R.M.Ș.); 2 Faculty of Food Engineering, Tourism and Environmental Protection; Institute for Research, Development and Innovation in Technical and Natural Sciences, “Aurel Vlaicu” University of Arad, 2 Elena Dragoi St., 310330 Arad, Romania; dana.copolovici@uav.ro (D.M.C.); pag.andreea@yahoo.com (A.L.); silvimot@yahoo.com (S.M.); lucian.copolovici@uav.ro (L.C.); 3 “Coriolan Dragulescu” Institute of Chemistry, 24 Mihai Viteazul Blvd., 300223 Timisoara, Romania; mdorosencu@yahoo.com; 4 Research Centre for Study of Food and Agricultural Products Quality, University of Agronomic Sciences and Veterinary Medicine of Bucharest, 59 Marasti Blvd., 011464 Bucharest, Romania; flstanica@yahoo.co.uk; 5 Faculty of Environmental Protection, University of Oradea, 26 Gen Magheru St., 410048 Oradea, Romania; svicas@uoradea.ro; 6 Biomedical Sciences Doctoral School, University of Oradea, 1 University St., 410087 Oradea, Romania

**Keywords:** DPPH assay, fruit morphology, genetic variability, solvent extracts, *Ziziphus* sp.

## Abstract

The genus *Ziziphus* includes numerous species, both cultivated and wild, offering significant genetic variability and economic potential that are often overlooked. Due to their high variability and ecological plasticity, jujube species and genotypes can be utilized in marginal areas and on land where few plants could be efficiently exploited. This study investigated variations in morphological characteristics (qualitative and quantitative), bioactive content (e.g., DPPH radicals), and antioxidant capacity in fruits, leaves, and stones of cultivated *Z. jujuba* genotypes (‘Hu Ping Zao’ and ‘Jun Zao’) and wild genotypes (*Z. acido-jujuba* and ‘Jurilovca’), using different solvents (water, ethanol, and methanol). The mass and dimensions of the fruits as well as their parameters (fresh and dry weight, length, width, and pulp-to-stone ratio) and the antioxidant potential of different plant organ types (leaves, fruit pulps, and stones) were determined. The results showed that the cultivated genotypes produced larger and heavier fruits with a higher pulp percentage than the wild forms of the same species. However, the wild forms exhibited higher antioxidant capacities than the cultivated genotypes, depending on the type of plant organ analyzed and the solvent used for extraction.

## 1. Introduction

The transition from wild to cultivated plant systems during the Holocene marked a crucial moment in the development of human civilization and terrestrial ecosystems [1]. However, this transition led to a strong focus on a limited number of major crops, while neglected and underutilized species (NUSs) were largely overlooked. These include cultivated genotypes, semi-domesticated plants, and wild species that are frequently insufficiently exploited both locally and globally due to their relatively low economic importance in global production and markets. They typically do not meet the standards of uniformity, color, and size met by major cultivated crops [2,3].

Traditionally, food security has focused on staple crops such as grains, roots, and tubers, which provide accessible sources of energy. In recent years, the focus has shifted towards nutritional security, highlighting the need for diverse nutrients for human health. The erosion of agricultural biodiversity represents a significant risk to food security by reducing nutrient diversity in diets [4].

The impacts of climate change add new challenges that underscore the importance of genetic diversity. Due to limited cultivated species, horticultural systems are highly vulnerable to extreme weather events. Increasing genetic diversity strengthens their resilience, protects against disruptions, and ensures both food security and the nutritional variety essential for human health [5].

In this context, NUSs play a crucial role. These species, often rich in vitamins, micronutrients, and phytochemicals, represent an underutilized resource that could contribute significantly to food security [2]. More widespread use of NUSs could not only improve the nutritional value of the food supply but also enhance agricultural systems’ adaptability and resilience to both biotic and abiotic stressors [6]. Many indigenous plant species offer high nutritional value compared to similar genotypes or species from the same family. Therefore, utilizing these plants would increase the potential to produce nutrient-dense foods. Most of these neglected plants (NPs) have a comparative advantage on marginal lands, where they have been naturally selected to withstand stressful conditions and, as a result, can contribute to sustainable production systems with reduced inputs [7].

For fruit-bearing trees and shrubs, NUSs demonstrate significant phytochemical potential, as shown by research on their health impacts in line with current trends in pharmacology and phytochemistry. Despite this potential, efforts to utilize these resources (such as NUSs and products with high antioxidant potency) are generally limited to regional levels or smaller commercial ventures [8,9].

Larger fruit sizes often characterize cultivated fruit species compared to their wild ancestors and relatives. For instance, cultivated jujubes produce significantly larger fruits (>10 g) compared to wild jujubes (<5 g) due to a higher pulp-to-stone ratio. Fruit size is determined by the final cell count and volume, which are significantly influenced by the intensity and duration of the cell division and expansion phases [10]. As important food resources, fruit trees provide essential nutrients such as proteins, amino acids, and vitamins. They are integrated into both traditional and modern agroecosystems worldwide, playing a crucial role in supporting human health and contributing to food security.

The Chinese jujube (*Ziziphus jujuba* Mill., syn. *Ziziphus sativa* Gaertn.), a member of the Rhamnaceae family, is commonly known as the “king of fruits” in arid regions or the “poor man’s apple.” This species was domesticated from wild jujube variants (*Z. jujuba* Mill. var. *spinosa*, *Ziziphus acido-jujuba*), which have evolved from small, sour fruits with a thin pericarp into larger, sweeter fruits with a larger proportion of edible pulp [11].

The wild sour jujube, also known as the wild jujube (*Z. acido-jujuba*; Rhamnaceae), is a species of significant economic and ecological value [12], known for its nutritious fruit, the medicinal importance of its stones, and its tolerance to drought and cold conditions [13]. It is the genetic ancestor of the cultivated jujube (*Z. jujuba* Mill.) and a valuable genetic resource for improving cultivated varieties.

Native to China, the species has been cultivated since ancient times and spread to Asia Minor, Europe, and America. This shrub grows rapidly with an expansive canopy, and its branches are characterized by thin, fluffy, brown spines arranged in pairs [14]. It is highly drought-tolerant due to its deep taproot system and xerophytic traits, such as thick bark and leathery leaves [15]. It grows well even in marginal or poor soils, where most other fruiting shrubs either fail to grow or perform poorly. Jujube stones contain saponins, jujuboside, and obelin lactone [16]. The wood is used as fuel or charcoal, while the leaves serve as feed for sheep and goats [17].

The jujube fruit is gaining popularity for its pleasant taste, unique aroma, and high nutritional value [18]. It is abundant with polysaccharides, amino acids, ascorbic acid, triterpenic acids, flavonoids, phenolic acids, and minerals. Studies suggest that jujube fruits have therapeutic potential due to their antioxidant, anti-inflammatory, antibacterial, and anticancer properties [19,20].

When fully ripe, the jujube fruit has soft, sweet pulp that is an excellent source of vitamin C (over 1000 mg/100 g fresh flesh), as well as vitamins A and B, carotenoids, proteins, minerals (Ca, P, K, Rb, Br, and La), and soluble sugars (fructose, glucose, and galactose) [21,22,23]. In addition to the nutritional and economic significance of the jujube, certain genotypes are utilized in landscaping due to the shape and color of the fruit and shrubs [24].

As the jujube market expands and consumer demand for high-quality, visually appealing fruit increases, selecting genotypes with desired fruit morphology and nutritional standards becomes imperative. The key morphological traits of the fruit include size, shape, pulp (mesocarp) weight, and stone weight, as well as the fruit-to-stone ratio. Significant variations in these traits have been across different jujube cultivars [25]. Quantitative traits, such as the size and weight of the fruit, directly affect the yield, while quality characteristics such as skin color, fruit shape, firmness (hardness), and the fruit-to-stone ratio are important for assessing fruit quality [26].

In Romania, wild populations of jujube have been found in the Dobrogea region [27], between the Danube River and the Black Sea, near ancient sites such as the Greek, Roman, and Byzantine ruins at Ostrov, Jurilovca, and Mahmudia. These ancient civilizations likely played a significant role in introducing this Asian plant to the area [28,29]. The fruits of these plants are consumed when ripe by birds and collected by the local population, either for fresh consumption or for producing alcoholic distillates through fermentation.

Therefore, the morphological characterization of jujube fruits is a valuable method for determining phenotypic diversity across species, forms, and genotypes. This research aimed to investigate variations in morphological characteristics (qualitative and quantitative), bioactive content (e.g., DPPH radicals), and antioxidant capacity in fruit, leaves, and stones using different solvents (water, ethanol, and methanol) in cultivated *Z. jujuba* genotypes (‘Hu Ping Zao’, ‘Jun Zao’) and wild genotypes (*Z. acido-jujuba* and ‘Jurilovca’). These analyses aimed to compare the productivity and quality parameters of bioactive compounds in cultivated and wild genotypes, highlighting their potential for use in breeding programs.

## 2. Results

### 2.1. The Morphological Traits of Fruits and Leaves

The morphological characteristics of fruits from various Ziziphus species and genotypes were evaluated through analysis of variance (F test), which revealed significant differences among genotypes. The greatest variability was observed in fruit weight, while the shape index exhibited the least variability (Table 1). Fruit length ranged from 16.3 mm in *Z. acido-jujuba* to 39.3 mm in ‘Jun Zao’. Compared to the wild genotypes, the cultivated ones yielded fruits of significantly greater length. Between the wild varieties, the fruit of *Z. acido-jujuba* was considerably shorter than that of ‘Jurilovca’. Regarding uniformity, cultivated genotypes exhibited enhanced consistency in fruit length within the intrapopulation context. Additionally, the width of cultivated fruits (26.1–26.7 mm) was notably larger than that of wild fruits (15.2–16.3 mm) and expressed moderate variability, particularly in ‘Jun Zao’. Fruit shapes in the cultivated genotypes were more elongated than in the wild type, with *Z. acido-jujuba* showing flatter fruits than ‘Jurilovca’. The fresh fruit weight varied between 1.73 g and 11.65 g, with significantly larger fruits observed in the cultivated genotypes, which varied among themselves. In this regard, *Z. acido-jujuba* exhibited lower fruit weight than ‘Jurilovca’ among the wild forms. Uniformity in fresh fruit weight was observed across all genotypes at the intrapopulation level, while dried fruit weights of cultivated genotypes were also significantly higher than those of wild genotypes.

The results presented in Table 2 indicate that genotype significantly affected pulp and stone characteristics. Regarding pulp weight, in both the fresh and dried states, cultivated genotypes were observed to have significantly higher values than wild ones, with ‘Hu Ping Zao’ showing the higher value of the two cultivated genotypes. Between the wild types, the fruits of the ‘Jurilovca’ genotype showed higher pulp weight. Moderate variability in fruit weight was observed in ‘Jun Zao’, while *Z. acido-jujuba* showed high uniformity. Furthermore, differences between wild and cultivated genotypes were also evident in stone weight, with significantly similar values observed for the wild genotypes.

Regarding the ratio between pulp and stone weight, a clear differentiation was observed among all genotypes. ‘Hu Ping Zao’ had a higher pulp ratio than both the wild genotypes and ‘Jun Zao’. The lowest pulp proportion in the fruit was found in *Z. acido-jujuba.*

**Figure 1:**
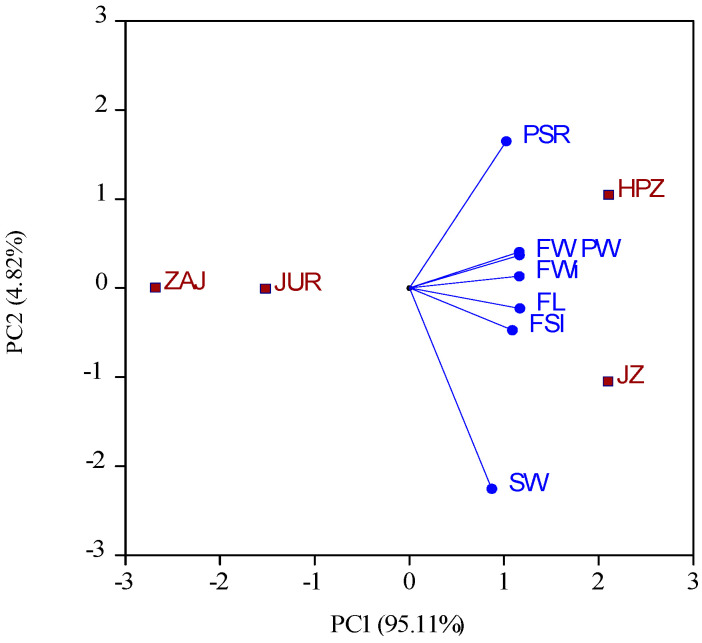
Biplot for fruit-, pulp-, and stone-related traits in jujube genotypes. HPZ-‘Hu Ping Zao’; JZ—‘Jun Zao’; ZAJ—Z. acido-jujuba; JUR—‘Jurilovca’; FL—fruit length; FWi—fruit width; FSI—fruit shape index; FW—fruit weight; PW—pulp weight; SW—stone weight; PSR—pulp-to-stone ratio.

The biplot based on the first two principal components (Figure 1), captures 99.93% of the variability in the traits analyzed for Chinese jujube genotypes. It clearly distinguishes between wild and cultivated genotypes.

Based on the variance components of multiple regression (Table S1), 96.7–99.72% of fruit weight variability in the four genotypes can be explained as the result of the influence of the five traits included in this regression model. In the cultivated genotypes, the pulp weight significantly impacted fruit weight (49.71–57.04%). Additionally, fruit length contributed 19.07–26.51% to fruit weight in these genotypes. For the wild genotypes, about 76.5% of fruit weight variation in Z. *acido-jujuba* could be attributed to the effects of fruit length, while in the ‘Jurilovca’ genotype, 93% of the fruit weight variation was due to pulp weight. The fruit shape index had the greatest impact on fruit weight in the ‘Hu Ping Zao’ genotype, while fruit width had the strongest influence in the ‘Jun Zao’ genotype. Regression analysis showed independent errors, as indicated by the Durbin–Watson (DW) coefficient for all genotypes. These results highlight clear differences in fruit morphology and components between cultivated and wild genotypes.

The analysis of correlations between fruit-, pulp-, and stone-related traits in jujube genotypes (Table S2) showed that in cultivated genotypes and ‘Jurilovca’, fruit weight was significantly positively correlated with pulp and stone weights, while in the case of *Z. acido-jujuba*, only the correlation between pulp weight and fruit weight was significant. For wild genotypes, fruit length and width were significantly correlated, indicating that their shapes differed from those of cultivated genotypes. In *Z. acido-jujuba*, fruit size was positively correlated with pulp and stone weights. In the cultivated ‘Jun Zao’ genotype, pulp and fruit weight increased with fruit width. Across all genotypes, fruit shapes were not correlated with fruit weight or its components.

**Figure 2:**
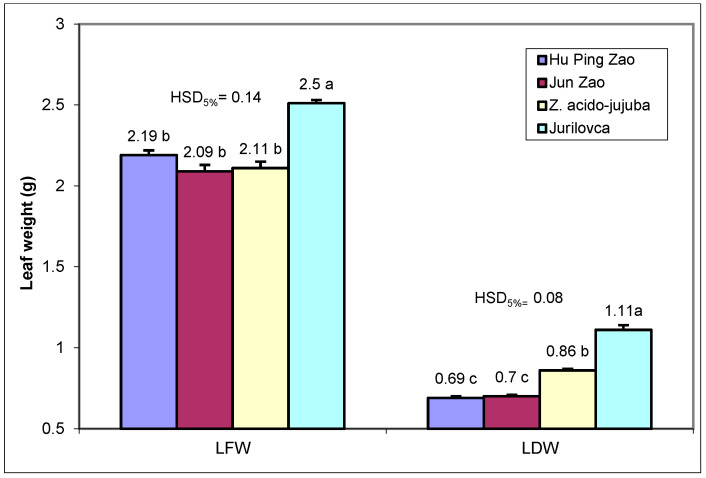
Fresh and dry weight of leaves from jujube genotypes. Different letters (a, b, and c) indicate significant differences between genotypes at *p* < 0.05 according to the Tukey test. LFW—Leaves fresh weight; LDW—Leaves dry weight.

The fresh and dry weight of leaves were significantly higher in the ‘Jurilovca’ genotype than in other genotypes (Figure 2). The dry leaf weight of the *Z. acido-jujuba* genotype was superior to the cultivated genotypes. Additionally, the dry matter content in the leaves of wild genotypes (42.2–44.4%) was higher compared to cultivated ones (31.5–33.5%).

To investigate how the morphological differences among the three organ types (leaves, pulp, and stone) of Ziziphus species affect their antioxidant properties, the DPPH radical scavenging activity (%) in different solvents was assessed.

### 2.2. Antioxidant Potential

Using ethanol as an extraction solvent, RSA (radical scavenging activity) values in the leaves of wild species were significantly higher by 12.11–19.46% compared to cultivated species (Table 3). Additionally, in the ‘Hu Ping Zao’ genotype, inhibition was higher by 13.16% compared to ‘Jun Zao’. The superiority of wild genotypes over cultivated ones was also observed in the RSA of fruit pulp, with significant differences among the genotypes of each group. For example, in *Z. acido-jujuba*, inhibition was significantly higher by 15.35% compared to ‘Jurilovca’, while for ‘Hu Ping Zao’, the value was higher by 12.42% compared to ‘Jun Zao’. In the case of stone extracts, RSA values among genotypes varied much less. Only the ‘Jurilovca’ genotype had significantly lower values, by 11.78–13.77%, compared to the other genotypes, which have similar values. In cultivated genotypes, significant variation in RSA was observed across the three organ types, with the stone showing higher inhibition than the pulp and leaf. In wild genotypes, RSA values for the stone and pulp were similar and significantly higher than for the leaf.

For methanol extracts, the genotypes showed low RSA variability in leaves, with Jun Zao exhibiting significantly lower inhibition compared to the other genotypes, which were similar to each other. For pulp extracts, RSA values varied significantly among genotypes, ranging from 25.95% for ‘Hu Ping Zao’ to 51.55% for ‘Jun Zao’, while wild forms displayed intermediate values between cultivated ones. Stone extracts exhibited an inhibition level of 25.09%, with higher RSA values in all other genotypes compared to ‘Jun Zao’. Significant differences between plant organs were noted in ‘Hu Ping Zao’ and ‘Jurilovca’, with values ranging from 25.95% in pulp to 73.22% in stones for ‘Hu Ping Zao’ and from 30.67% in leaves to 65.59% in stones for ‘Jurilovca’. For ‘Jun Zao’, leaf RSA was significantly lower, while *Z. acido-jujuba* showed significantly higher stone RSA.

Aqueous leaf extracts exhibited inhibition ranging from 3.51% in ‘Jun Zao’ to 29.96% in *Z. acido-jujuba*, with wild genotypes showing significantly higher values (11.95–26.45%) compared to cultivated ones, and minimal variation within each group. In aqueous pulp extracts, genotype had no significant effect on RSA. However, in both cultivated genotypes and *Z. acido-jujuba*, RSA from pulp extracts was significantly higher than from leaf extracts.

**Figure 3:**
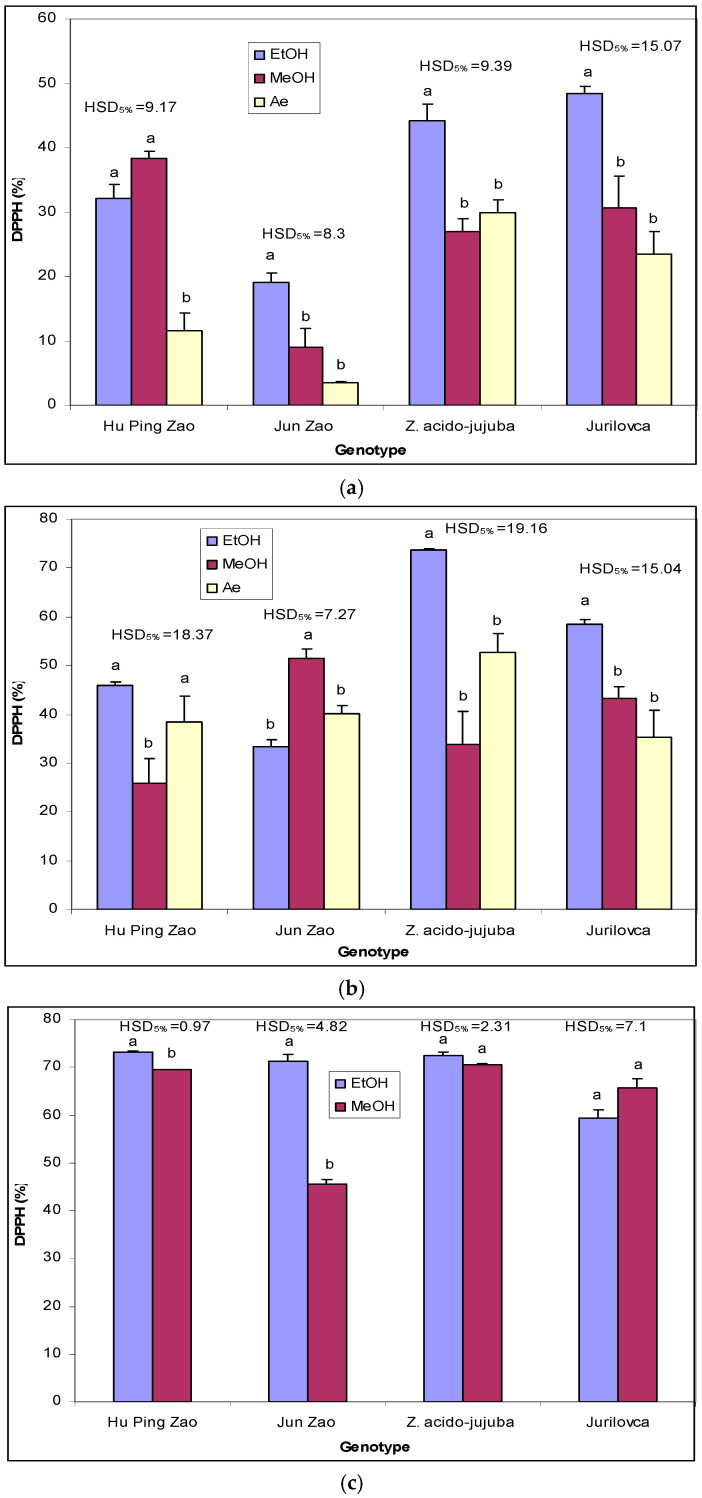
The effect of extraction solvents on DPPH radical scavenging activity (%) in the leaves (**a**), pulp (**b**), and stones (**c**) of jujube genotypes. EtOH—ethanol extract; MeOH—methanol extract; Ae—aqueous extract. Different letters indicate significant differences between solvents at *p* < 0.05 according to the Tukey test.

For leaves, using ethanol and methanol as extraction solvents in the ‘Hu Ping Zao’ genotype showed similar efficiency, yielding significantly higher RSA values than aqueous extracts (Figure 3a). In other genotypes, ethanol extracts demonstrated significantly higher inhibition. For pulp extracts, methanol had a significantly lower effect on RSA in ‘Hu Ping Zao’ but was substantially more effective in ‘Jun Zao’ (Figure 3b). In wild genotypes, ethanol extracts from pulp had the highest RSA values. Stone extract inhibition was significantly influenced by the solvent only in wild genotypes, wherein ethanol proved more effective than methanol (Figure 3c).

Ethanol extracts of leaves showed significant variation in antioxidant capacity, ranging from 3.48 μmol TE/g DW in ‘Jurilovca’ to 13.07 μmol TE/g DW in ‘Hu Ping Zao’, with *Z. acido-jujuba* having higher values than ‘Jun Zao’ (Table 4). Wild genotypes had significantly higher antioxidant capacity in pulp extracts compared to cultivated genotypes, with *Z. acido-jujuba* recorded as having the highest values and ‘Jun Zao’ the lowest. In stone extracts, the variation in antioxidant capacity among genotypes was minimal, with only ‘Jurilovca’ showing a significantly lower value than the others, which were relatively similar.

When methanol was used as the extraction solvent, the leaves of the ‘Hu Ping Zao’ genotype showed significantly higher antioxidant capacity compared to the other cultivars, followed by *Z. acido-jujuba*, which had significantly higher values than ‘Jun Zao’ and ‘Jurilovca’. The effect of genotype on the antioxidant capacity of pulp extracts was less pronounced, with Jun Zao and Jurilovca showing significantly higher values than ‘Hu Ping Zao’. For stone extracts, antioxidant capacity ranged from 1.68 μmol TE/g DW in ‘Jun Zao’ to 2.62 μmol TE/g DW in *Z. acido-jujuba*, with ‘Hu Ping Zao’ and *Z. acido-jujuba* showing significantly higher values than the other two genotypes.

Aqueous extracts from *Z. acido-jujuba* leaves exhibited the highest antioxidant capacity, followed by ‘Hu Ping Zao’, while ‘Jun Zao’ and ‘Jurilovca’ had the lowest values. In pulp extracts, the variation among genotypes was considerably lower, with only *Z. acido-jujuba* showing significantly higher antioxidant capacity than the other genotypes, which had similar values.

**Figure 4:**
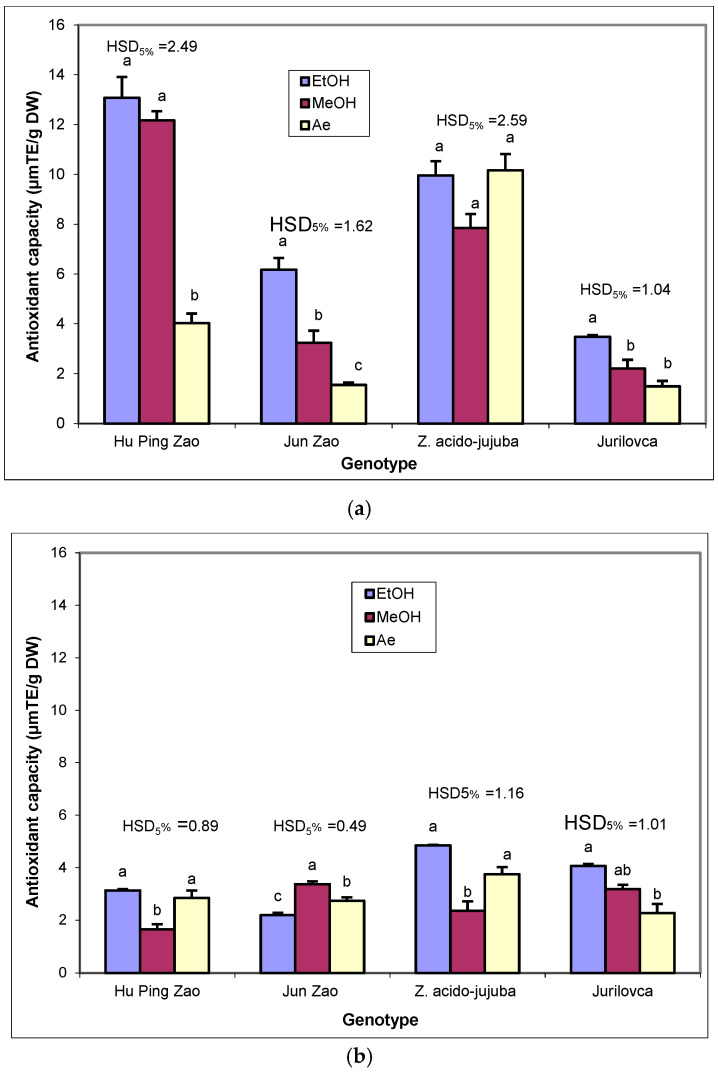

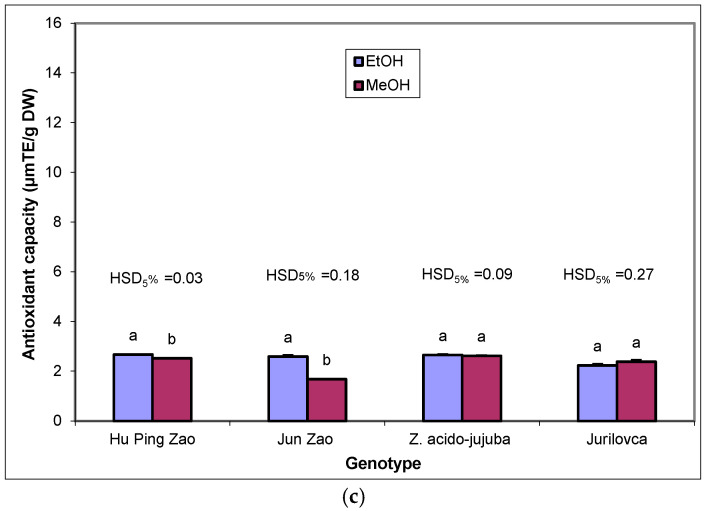
The effect of extraction solvents on antioxidant capacity (μmol TE/g D) in the leaves (**a**), pulp (**b**), and stones (**c**) of jujube genotypes. EtOH—ethanol extract; MeOH—methanol extract; Ae—aqueous extract. Different letters indicate significant differences between solvents at *p* < 0.05 according to the Tukey test.

The effect of solvents on the antioxidant capacity of leaf extracts (Figure 4a) varied according to the genotype. In ‘Hu Ping Zao’, ethanol and methanol showed similar efficacy, significantly higher than that of water. The solvent influence was strongest for ‘Jun Zao’, with significant differences among the three solvents, ethanol extract having the highest value and aqueous extract the lowest. For the wild genotype *Z. acido-jujuba*, the antioxidant capacity of the leaves was not significantly affected by the solvents. In ‘Jurilovca’, ethanol had a greater effect on the antioxidant capacity than methanol and aqueous extracts.

The solvent choice showed similar effects on the antioxidant capacity of the pulp extracts in ‘Hu Ping Zao’ and *Z. acido-jujuba*, with significantly higher values for the ethanol and aqueous extracts compared to the methanol extract (Figure 4b). The strongest solvent effect was observed for ‘Jun Zao’, with significant differences between the three solvents, the values being higher for methanol and lower for ethanol. In ‘Jurilovca’, the use of ethanol as a solvent resulted in a significantly higher antioxidant capacity compared to the use of water. For stone extracts, ethanol had a higher antioxidant capacity than methanol only in the cultivated ‘Jun Zao’ genotype (Figure 4c).

The ethanol extract of leaves expressed significant higher FRAP values in wild genotypes compared to cultivated genotypes, on the background of a significant increase in ‘Jun Zao’ compared to ‘Hu Ping Zao’ (Table 5). The same superiority of FRAP values in wild genotypes was also observed in the case of pulp extract. Amid a low variation in FRAP in the stone extract, only the value of ‘Hu Ping Zao’ was significantly lower compared to other genotypes. In the case of wild genotypes, the extract from leaves exhibited higher FRAP than the extracts from pulp and stones. For the ‘Hu Ping Zao’ genotype, the extract from leaves had a significantly higher FRAP value compared with those from pulp and stone, which had close values. In the ‘Jun Zao’ genotype, significant variation in FRAP was observed between the plant organs, with the highest value for leaf extract and the lowest value for pulp extract.

Using methanol as an extraction solvent, the FRAP values from leaves ranged from 16.25 mM Trolox/L in *Z. acido-jujuba* to 18.95 mM Trolox/L in the ‘Jurilovca’ genotype, which indicated a significant variation between genotypes. The FRAP values among genotypes varied much less in pulp extract. As such, only *Z. acido-jujuba* had a significantly lower value compared to the other genotypes, which had close values. The cultivated genotypes expressed significantly higher FRAP values of stone extracts compared to the wild genotypes. Significant differences between FRAP from different plant organs were noted in ‘Jun Zao’ and ‘Jurilovca’, with the highest value in leaves, and in *Z. acido-jujuba*, with the highest value in stone extract. In ‘Hu Ping Zao’, the leaf extract exhibited higher FRAP compared with the close values of the pulp and stone extracts.

Aqueous leaf extracts exhibited FRAP values ranging from 16.76 mM Trolox/L in ‘Jun Zao’ to 17.56 mM Trolox/L in ‘Hu Ping Zao’, with wild genotypes showing intermediate values compared to cultivated ones. In aqueous pulp extracts, the genotype had a smaller significant effect on FRAP, given that only the value for *Z. acido-jujuba* was significantly lower, while the other genotypes had similar values. For all genotypes, the FRAP value of leaf extracts was significantly higher than that of pulp extracts.

**Figure 5:**
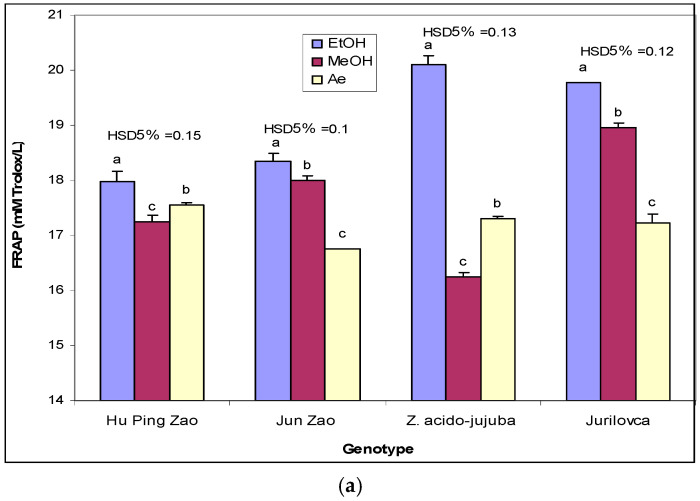

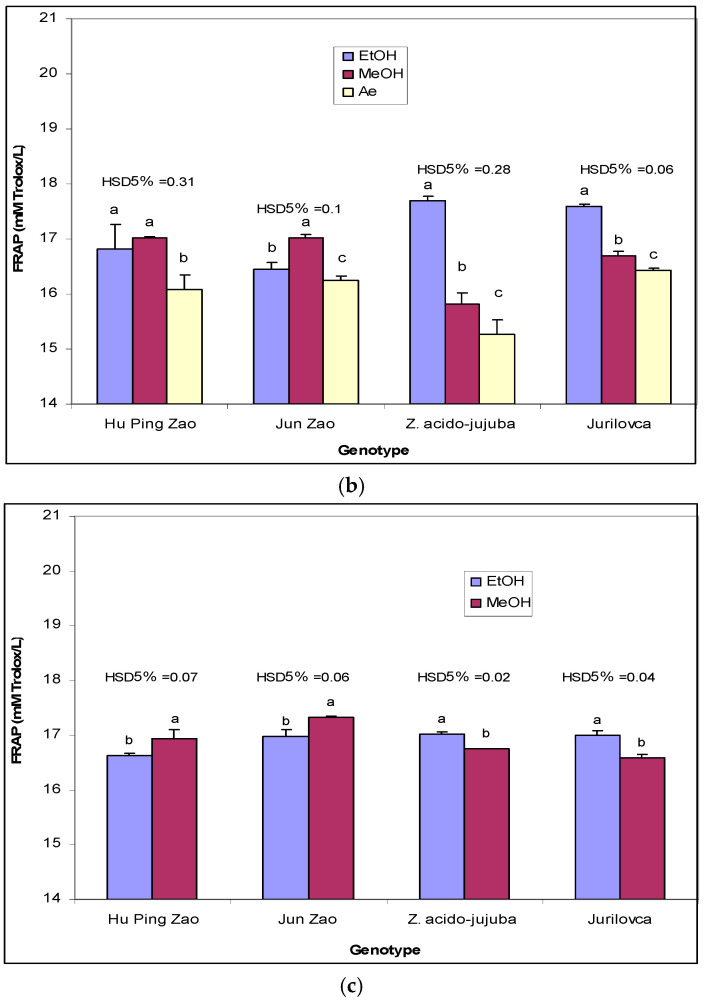
The effect of extraction solvents on FRAP (μM TE/L) in leaf (**a**), pulp (**b**), and stone (**c**) of jujube genotypes. EtOH—Ethanol extract; MeOH- Methanol extract; Ae—Aqueous extract. Different letters indicate significant differences between solvents at *p* < 0.05 according to the Tukey test.

For all genotypes, the solvent used had a significant effect on the FRAP of leaf extract, with the highest value for ethanol (Figure 5a). In the cases of the ‘Jun Zao’ and ‘Jurilovca’ genotypes, the methanol extracts showed significantly higher FRAP values than the aqueous extracts, while in ‘Hu Ping Zao’ and *Z. acido-jujuba*, the ethanol extracts demonstrated significantly lower FRAP. In pulp samples, the methanol extract had a higher FRAP for the cultivated genotypes, while for wild genotypes, the superiority of ethanol extracts was obvious (Figure 5b). In the case of stone samples, the FRAP values of ethanol extracts were significantly higher in wild genotypes, while for cultivated genotypes, the methanol extracts presented the best FRAP values (Figure 5c).

**Figure 6:**
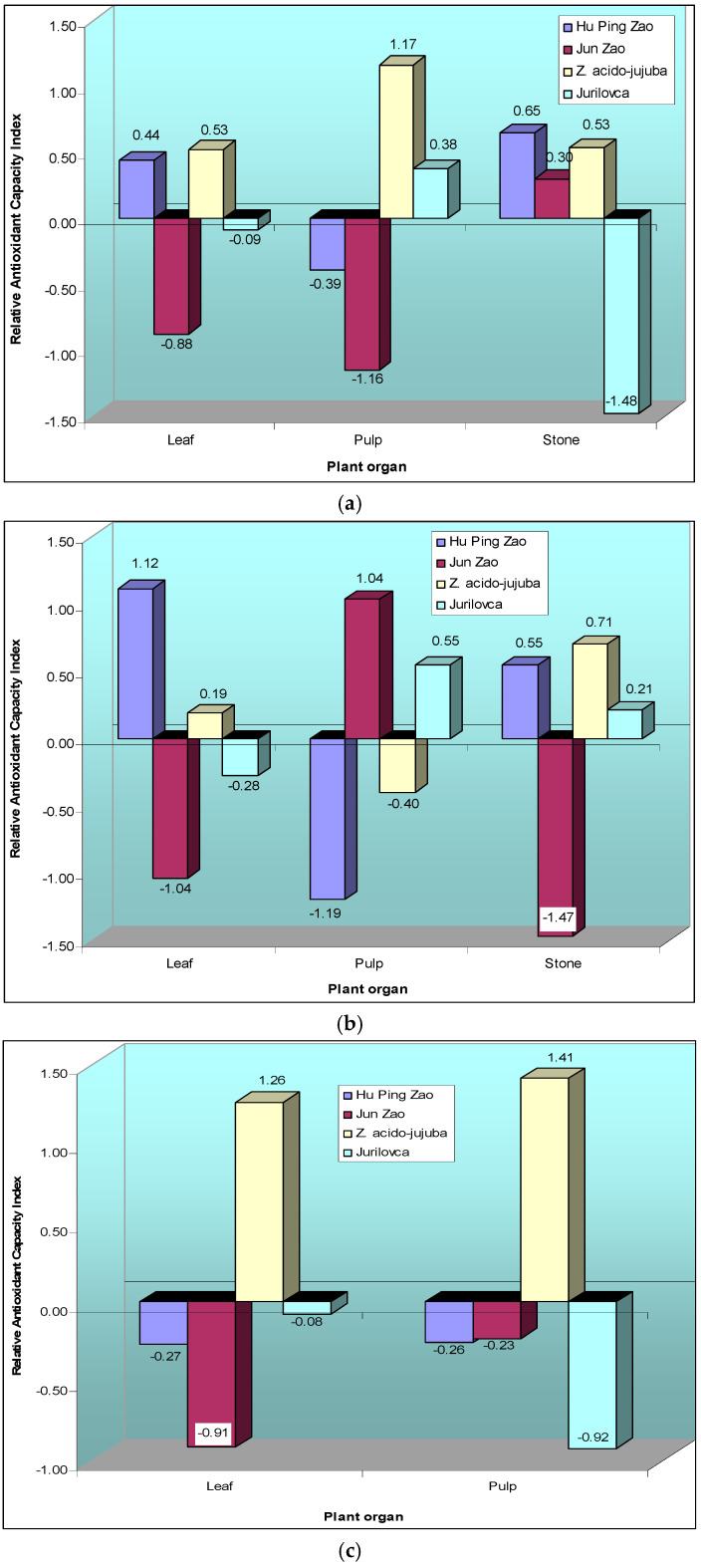
Relative antioxidant capacity index values for the leaves, pulp, and stones of jujube genotypes using different extraction solvents (EtOH—(**a**); MeOH—(**b**); Ae—(**c**)).

Based on the relative antioxidant capacity index (RACI), the ethanolic leaf extracts of *Z. acido-jujuba* and ‘Hu Ping Zao’ showed the highest antioxidant capacity (Figure 6a). For pulp extracts, the wild genotypes, particularly *Z. acido-jujuba*, showed superior antioxidant capacity. In the case of stone extracts, all genotypes except Jurilovca showed higher antioxidant capacity than cultivated varieties.

When methanol was used as the solvent, the leaf extracts of the ‘Hu Ping Zao’ genotype showed the highest antioxidant capacity, while the ‘Jun Zao’ extracts had significantly lower values (Figure 6b). For pulp extracts, the ranking of genotypes changed, with ‘Jun Zao’ having the highest antioxidant capacity and ‘Hu Ping Zao’ having the lowest. In stone extracts, ‘Jun Zao’ exhibited notably lower antioxidant capacity than the other genotypes. In the aqueous extracts, the wild genotype *Z. acido-jujuba* exhibited significantly higher antioxidant capacity in both pulp and leaves (Figure 6c).

The data in Table S3 revealed significant correlations between the extraction solvents regarding the ranks of jujube genotypes for DPPH radical scavenging activity of leaf and stone extracts. The ranking of the four jujube genotypes regarding the antioxidant capacity was not significantly affected by the solvent used for extraction, according to the positive and significant correlations shown in Table S4.

The first two principal components shown in Figure S1 fully express the DPPH radical scavenging activity in different plant organs of jujube genotypes using three extraction solvents. Given its positive PC1 value, the ethanol extract exhibits the highest DPPH radical scavenging activity for most combinations of genotype and plant organ, except for the pulp of the ‘Jun Zao’ genotype. As such, ethanol can be considered the most effective for antioxidant extraction, as measured by DPPH radical scavenging activity, from the pulp, stone, and leaves of jujube. Water has the lowest extraction efficiency for the assessment of DPPH radical scavenging activity for most combinations of genotype and plant organ, while in the leaves and pulp of *Z. acido-jujuba* and the pulp of ‘Hu Ping Zao’, methanol expressed the lowest extraction efficiency.

According to the biplot in Figure S2, the first principal component contributed 86.77% of the total variance of antioxidant capacity in different plant organs of jujube genotypes using three extraction solvents, while the contribution of the second principal component was 13.23%. Considering its positive PC1 and PC2 values, the ethanolic extract showed the highest antioxidant capacity for most of the genotypes and plant samples. In the case of the pulp of the Jun Zao genotype, methanol was the most effective for the extraction of antioxidant compounds. For samples of the leaves and pulp of *Z. acido-jujuba* and the pulp of ‘Hu Ping Zao’, the values of the aqueous extract were higher than those of the methanolic extract.

The variation in FRAP in different plant organs of jujube genotypes using three extraction solvents can be fully expressed by a biplot based on the first two principal components (Figure S3). The ethanolic extract showed a strong association with the PC1 axis, which makes a major contribution to the analysis of FRAP variation. According to its projections on the vectors of different analyzed samples, the ethanol extract exhibited the highest FRAP for all combinations of genotype and plant organ, except for the pulp of the ‘Jun Zao’ genotype, where the methanol was the most effective.

## 3. Materials and Methods

### 3.1. Biological Material

Fruits and leaves from four jujube genotypes were used for analysis; specifically, the breed genotypes Hu Ping Zao and Jun Zao and the Jurilovca landrace of the species *Z. jujuba* were used, as was the wild species *Z. acido-jujuba.*

**Figure 7:**
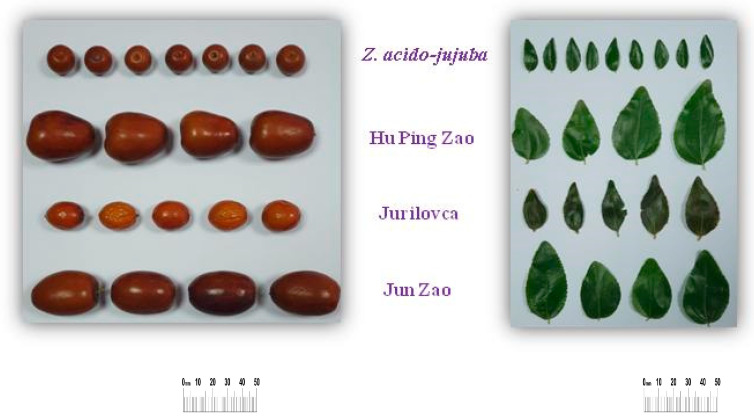
Morphology of fruits and leaves in the studied jujube genotypes.

The biological samples were obtained from the collection of the Department of Fruit Growing of the University of Agronomic Sciences and Veterinary Medicine in Bucharest (*Z. acido-jujuba* sp. cv. ‘Hu Ping Zao’ and cv. ‘Jun Zao’) and collected from nature in the area of Jurilovca Village (44°48′50.33″ N/28°50′16.75″ E) (‘Jurilovca’ landrace). Fruit and leaf harvesting took place from three mature plants with fully ripe fruit (brownish-red coloration on the entire fruit surface) (Figure 7).

The experiment followed a completely randomized design and included three replicates. Each replicate, including 5 fruits and leaves, was used for the physical analysis of fruit weight, length, width, fresh and dry weight, pulp and stone fresh and dry weight, fresh leaves, and dry weight. The shape index (fruit length/width) and pulp/stone ratios were calculated based on these measurements. Additionally, 20 more fruits (including pulp and stones) and leaves were dried and ground for biochemical analyses, including solvent extraction and antioxidant capacity assessment (DPPH assay).

The masses of the fruits and their components were measured using a precision analytical balance, while the dimensions of the fruits were recorded with an electronic caliper. The moisture content of the fruit samples was determined using a drying oven (Thermo Fisher Scientific Inc., Asheville, NC, USA) at 70 °C until a constant dry weight was obtained. The percentage moisture content was calculated using the following formula:

Moisture (%) = [(initial weight − final weight)/initial weight] ×100

### 3.2. Sample Preparation

A homogeneous powder was obtained by grinding the dried leaves and fruit flesh with a mill. The powder was then extracted with three different solvents (water, 90% ethanol, and 90% methanol) at a 1:10 (*w*/*w*) ratio. The stones were first degreased with hexane for 2 h, dried, and then extracted using the same three solvents. The extracts were filtered through a 0.45 µm membrane filter and stored in the fridge until analysis.

### 3.3. Antioxidant Capacity Determined by DPPH Assay

The DPPH (2, 2-Diphenyl-1-picrylhydazyl) assay was performed according to the method of Lee et al., 2013 [1] with some modifications. Briefly, each sample or standard (Trolox) was added to 0.2 mM DPPH at a ratio of 1:4 (*v*/*v*) in sterile polystyrene 96-well microplates (Corning Inc., Life Sciences, Corning, NY, USA) and then incubated at 37 °C for exactly 30 min in the dark. The absorbance was measured at 492 nm using a Stat Fax 2100 microplate reader (Fisher Bioblock Scientific, Fair Lawn Industrial Park, NJ, USA). A linear calibration curve was generated based on Trolox solutions with concentrations ranging between 0.015 and 0.6 mM (y = 3349.1x − 0.1589, R^2^ = 0.9983), and the results were expressed as µmol Trolox equivalent (TE)/g. The DPPH radical scavenging activity (%) was calculated using Formula (1):

DPPH radical scavenging activity (%) = [1 − (A_0_/As)] × 100 (1)

where A_0_ is the absorbance of the blank and As is the absorbance of the sample or standard.

### 3.4. Antioxidant Capacity Determined by Ferric Reducing Antioxidant Power (FRAP) Assay

The FRAP reagent was prepared by taking acetate buffer (3.6 pH), 10 mmol of TPTZ dissolved in 40 mmol of hydrochloric acid (HCl), and 20 mmol of dissolved iron (III) chloride in a 10:1:1 (*v*/*v*) ratio. Then, a 200 µL sample was added to the FRAP reagent (1.5 mL). The absorbance was measured at 593 nm after 20 min in dark, and the results were expressed in mM Trolox/L.

### 3.5. Statistical Analysis

The data from all analyses were statistically processed using ANOVA, and means were compared using Tukey’s HSD (Honestly Significant Difference) test [30]. Means with different letters are considered significantly different (*p* < 0.05). Relationships between fruit, pulp, and stone traits were analyzed using the Pearson and Spearman correlation coefficients and multiple regressions [30]. The significance of correlation coefficients was analyzed using the two-tailed test. A biplot based on the first two principal components for fruit, pulp, and stone traits was generated using BioVinci 1.5.1.

To consolidate the DPPH radical scavenging and antioxidant activity data into a single value, the Relative Antioxidant Capacity Index (RACI) was used, as described by Sun and Tanumihardjo, 2007 [31]. This index represents the mean of standard scores transformed from the initial data.

## 4. Discussions

Phenotypic diversity research in plants plays a key role in evaluating, conserving, and utilizing genetic resources, helping to determine the uniqueness and distinctiveness of genotypes. This information is essential for crop improvement [32]. Morphological analysis provides valuable insights for identifying relationships among species within the same botanical family and contributes to the advancement of knowledge for breeding programs. Additionally, understanding the morphological traits of jujube fruits facilitates the development of high-quality commercial varieties with enhanced tolerance to stress factors. Thus, morphological characterization is a fundamental step in identifying valuable genetic resources [33,34].

Morphological variations in jujube fruits can be attributed to a range of factors, including genetic, geographic, climatic, and ecological influences, as well as cross-pollination, natural hybridization, gene flow within the species, and human intervention [34,35].

The results confirm the significant intra- and interspecific variability within the *Ziziphus* genus, especially between cultivated genotypes and wild forms. Morphological analysis of the fruits indicated that fruit weight exhibited the highest variability, whereas the shape index showed the lowest. Wild and spontaneous forms demonstrated considerably smaller fruit dimensions and weights compared to cultivated genotypes. These results align with other studies [25,34,36], which established a strong relationship among fruit morphology, species, and the level of cultivar improvement.

Fruit weight generally ranged between 10 and 30 g, with some variations: 2.90–28.99 g/fruit [36], 14.26–32.69 g/fruit [37], and 4.71–39.02 g/fruit in *Ziziphus mauritiana* Lamk. [38]. Notably, some cultivated genotypes were reported to have fruit weights exceeding 50 g/fruit [39].

In this study, fruit length and diameter ranged from 14.3 to 39.3 mm and 15.2 to 26.0 mm, respectively, with wild forms displaying smaller dimensions. Previous studies on ancestral *Ziziphus* forms reported fruit lengths not exceeding 25 mm and irregular pulp thickness [40,41], cited by [36]. In contrast, cultivated genotypes show greater dimensions, with fruit lengths and diameters of 23–44.75 mm and 20.96–37.31 mm, respectively [38]. Additional studies have demonstrated variability across *Ziziphus* species, with lengths and diameters of 9.51–16.52 mm and 9.20–23.26 mm in *Z. jujuba*, 31.21–45.33 mm and 26.45–39.61 mm in *Z. mauritiana*, 11.16–17.72 mm and 11.88–18.43 mm in *Z. spina-christi*, and 10.32–15.4 mm and 12.22–16.3 mm in *Z. nummularia* [34]. These findings highlight the significant morphological variability of fruits within the *Ziziphus* genus, both among species and within genotypes of the same species, reflecting the influence of genetic and environmental factors.

The shape index of jujube fruit, defined as the ratio of diameter to length, is considered an important trait for quality assessment and genetic identification [38,42]. In this study, the shape index ranged from 0.9 to 1.51, indicating variations from round to oval shapes, with lower values observed in wild forms and higher values in cultivated genotypes. Other studies reported shape index values ranging from 0.6 to 0.91 [38], 1.04 to 2 [36], 1.08 to 1.39 [43], and 0.76 to 1.05 [44].

For fresh and dry pulp and stone weights, our results showed that cultivated genotypes had significantly higher values than wild forms, with the highest values recorded in the Hu Ping Zao genotype. The fresh pulp-to-stone ratio ranged from 3.22 in *Z. acido-jujuba* to 21.01 in Hu Ping Zao (Table 2), demonstrating significant variability between wild species and cultivated genotypes. These results align with those of Khadivi et al. [45], Khadivi and Beigi [46], Mirheidari et al. [47], Khadivi [34], and Sharif et al. [48], who reported notable morphological differences in fruit components across various *Ziziphus* species and genotypes. Therefore, the size and pulp-to-stone ratio serve as essential indicators for evaluating the domestication level of woody fruit species [49,50].

Morphological analysis of fruit parameters revealed significant differences between contemporary cultivated jujube genotypes and their ancestral wild forms. Cultivated jujube stones are generally smaller than those of wild forms, a characteristic potentially linked to domestication, suggesting the possibility of parallel evolution between *Z. jujuba* and *Z. acido-jujuba*. Studies show that the size and shape of jujube stones are primarily influenced by fruit dimensions, with strong correlations observed between fruit size, weight, and the pulp-to-stone ratio. During domestication, the increase in stone length led to a corresponding enlargement of the mesocarp size, thus enlarging the edible portion of the fruit [50,51,52].

Wild and cultivated jujube varieties differ not only in their morphological parameters but also in their biochemical properties, including RSA activity and antioxidant capacity. Antioxidants are essential in neutralizing free radicals and possess anti-inflammatory and anticancer properties. Recent studies have emphasized the significant antioxidant potential of phenolic compounds in jujube fruits and leaves [43,53]. Furthermore, various *Ziziphus* species are abundant with phytochemicals such as flavonoids, phenols, triterpenoids, and alkaloids [54,55,56]. Our results align with observations that biochemical composition of jujube is influenced by factors such as species characteristics [43,57], genotype [58], plant organ [44], extraction methods [59], and the specific environmental conditions of the region [44,60].

This study shows the superior antioxidant potential of wild jujube genotypes compared to cultivated ones, suggesting that challenging environmental conditions may contribute to their physiological and morphological adaptation. In wild genotypes, significantly higher RSA values in leaves and fruit pulp compared to cultivated genotypes indicate a potentially better resistance to oxidative stress. In cultivated genotypes, RSA variability is greater across plant organs (with the stone showing the highest activity), while in wild genotypes, both the stone and the pulp have higher values than the leaves. These findings suggest that wild genotypes may have greater potential for nutritional or therapeutic uses due to their increased antioxidant activity.

Additionally, the extraction solvent significantly influenced the antioxidant potential across different jujube plant organs. Generally, ethanol and methanol extracts showed superior antioxidant activity for both wild and cultivated genotypes. Aqueous extracts, on the other hand, displayed less variability in antioxidant activity, reflected in low concentrations of bioactive compounds such as polyphenols and flavonoids. However, the effect of the solvent varied depending on the genotype and the plant organ. For example, in *Z. acido-jujuba* leaves, the solvent type did not significantly affect antioxidant capacity, whereas for *Jun Zao*, ethanol yielded the best results, and water had the least effect. For the wild genotype *Z. acido-jujuba*, the antioxidant capacity of the leaves was not significantly affected by the type of solvent, whereas for Jurilovca, ethanol had a stronger effect on antioxidant capacity compared to methanol or water as a solvent. In *Ziziphus* leaves, flavonoid content is concentrated in the cuticle and epidermis, resulting in higher antioxidant capacity compared to the fruit. This statement is partially supported by our findings, as it depends on the type of solvent used for extraction, as well as the species and genotype. Our results show that leaves exhibited higher antioxidant capacity than pulp or stones when extracted with alcoholic solvents in both wild species and cultivated genotypes, while in cultivated genotypes, activity was more evenly distributed across organs. These findings align with previous studies [43,44,61]. However, when using aqueous solvents, the extracts obtained from fruit pulp displayed significantly higher antioxidant activity than those from leaves. Thus, the antioxidant capacity of *Ziziphus* leaves and fruit components varies considerably and is significantly influenced by the type of solvent, species, and genotype. Significant differences in antioxidant capacity across various plant organs and solvents suggest the importance of solvent, species, and genotype in harnessing the antioxidant potential of jujube.

Hence, the efficiency of the two solvent extraction methods (DPPH and FRAP) depends on the nature of the plant substrate as well as the type of extractable compounds. The correct choice of solvent leads to an increase in the extraction yield of antioxidants from the tissue. The results of this study highlight the above, as each type of solvent generated different results. Thus, alcoholic extracts, especially ethyl extracts, yielded better results compared to methyl or aqueous extracts in all four genotypes and three tissues (organs). In all extracts, water had lower efficiency than alcoholic solvents. A direct connection was observed between the total phenolic content and antioxidant activity, determined by the DPPH and FRAP assays.

## 5. Conclusions

Significant differences in both morphological traits and antioxidant capacity were identified among the studied jujube species, plant organ types, genotypes, and extraction methods. These observations reveal the genetic diversity within the *Ziziphus* genus, particularly between wild and cultivated genotypes. A detailed comparison of fruit morphology showed that cultivated fruits were larger, heavier, and more elongated and had a higher pulp-to-stone ratio than fruits of wild genotypes.

The analysis of antioxidant potential showed higher antioxidant values in wild genotypes, especially in leaves and pulp, regardless of the solvent used. Significant variation was also noted among cultivated genotypes, which demonstrated advantages in specific organs, such as stones, which showed the highest RSA values in most cases. The choice of extraction method influenced antioxidant potential, with ethanol and methanol extracts providing higher antioxidant potential than aqueous extracts. As such, ethanol can be utilized to produce extracts from fruits and leaves of jujube with enhanced antioxidant properties.

These results confirm that both the morphological features of fruits and leaves, as well as their antioxidant potential, are influenced by genetic factors, species, genotype, and the specific extraction methods used. Therefore, the results confirm the hypothesis that the morphological traits of fruits and leaves, as well as their antioxidant capacity, are strongly genetically determined. These characteristics depend on the species and genotype, as well as on the methods used for phenolic extraction.

## Figures and Tables

**Table 1:** Variation in fruit-related traits of jujube genotypes.

Genotype	Fruit Length (mm)		Fruit Width (mm)		Fruit Shape Index		Fruit Fresh Weight (g)		Fruit Dry Weight (g)	
Hu Ping Zao (*Z. jujuba)*	37.4 ± 0.8 a		26.70 ± 1.16 a		1.42 ± 0.07 a		11.65 ± 0.32 a		3.76 ± 0.07 a	
Jun Zao (*Z. jujuba)*	39.3 ± 0.5 a		26.10 ± 0.66 a		1.51 ± 0.04 a		10.32 ± 0.31 b		3.56 ± 0.11 a	
*Z. acido-jujuba*	16.3 ± 0.4 c		15.20 ± 0.53 b		0.90 ± 0.02 c		1.73 ± 0.03 d		0.74 ± 0.01 c	
Jurilovca (Z. *jujuba*)	20.2 ± 0.8 b		16.30 ± 0.65 b		1.24 ± 0.03 b		3.43 ± 0.06 c		1.40 ± 0.02 b	
F	396.19 **		60.99 **		42.83 **		464.98 **		532.03 **	
HSD_5%_	2.4		3.1		0.16		0.87		0.25	
Genotype	CV (%)	Range	CV (%)	Range	CV (%)	Range	CV (%)	Range	CV (%)	Range
Hu Ping Zao	6.45	33–40	13.80	21–33	14.97	1.15–1.90	8.76	10.14–13.2	5.56	3.32–4.08
Jun Zao	4.33	36–42	7.97	22–28	7.48	1.41–1.77	9.73	8.66–11.24	9.92	2.96–4.06
*Z. acido-jujuba*	9.30	11–15	11.10	11–17	5.87	0.81–1.01	4.65	1.58–1.83	2.49	0.71–0.76
Jurilovca	12.08	16–24	12.62	13–21	7.28	1.12–1.31	5.50	3.05–3.61	5.33	1.3–1.52

Different letters (a, b, c, d) in the same column indicate significant differences between genotypes at *p* < 0.05 according to the Tukey test. ** significant at *p* < 0.01 according to F test.

**Table 2:** Variation in pulp- and stone-related traits of jujube genotypes.

Genotype	Pulp Fresh Weight (g)		Pulp Dry Weight (g)		Stone Fresh Weight (g)		Stone Dry Weight (g)		Pulp: Stone Ratio (FW)		Pulp: Stone Ratio (DW)	
Hu Ping Zao	10.91 ± 0.28 a		3.50 ± 0.07 a		0.52 ± 0.02 b		0.26 ± 0.01 b		21.01 ± 0.31 a		13.47 ± 0.57 a	
Jun Zao	9.51 ± 0.25 b		3.11 ± 0.1 b		0.81 ± 0.01 a		0.44 ± 0.01 a		11.76 ± 0.32 b		7.02 ± 0.15 b	
*Z. acido-jujuba*	1.33 ± 0.01 d		0.45 ± 0.01 d		0.42 ± 0.01 c		0.28 ± 0.01 b		3.22 ± 0.05 d		1.60 ± 0.02 d	
Jurilovca	2.98 ± 0.05 c		1.13 ± 0.02 c		0.45 ± 0.01 c		0.27 ± 0.01 b		6.63 ± 0.13 c		4.21 ± 0.11 c	
F	453.93 **		559.16 **		219.08 **		117.4 **		110.19 **		292.81 **	
HSD_5%_	0.85		0.24		0.05		0.03		0.88		1.14	
Genotype	CV (%)	Range	CV (%)	Range	CV (%)	Range	CV (%)	Range	CV (%)	Range	CV (%)	Range
Hu Ping Zao	9.03	9.53–12.54	6.17	3.06–3.82	10.45	0.44–0.62	11.06	0.23–0.32	4.61	19.69–22.42	13.29	10.25–15.61
Jun Zao	10.38	7.89–11.18	10.53	2.54–3.59	2.72	0.77–0.84	7.05	0.39–0.48	8.58	10.25–13.63	6.65	6.0–7.61
*Z. acido-jujuba*	3.48	1.26–1.39	3.53	0.42–0.47	5.71	0.37–0.45	3.14	0.26–0.3	5.08	2.98–3.43	4.21	1.51–1.71
urilovca	5.46	2.66–3.14	5.26	1.03–1.21	9.43	0.39–0.53	9.01	0.23–0.31	6.34	5.92–7.37	8.52	3.81–5.04

Different letters (a, b, c, d) in the same column indicate significant differences between genotypes at *p* < 0.05, as determined by the Tukey test. ** significant at *p* < 0.01 according to F test.

**Table 3:** DPPH radical scavenging activity (%) of the leaves, pulp, and stones of jujube genotypes using different extraction solvents.

Extraction	Genotype	Plant Organ			HSD_5%_
Solvent		Leaf	Pulp	Stone	
EtOH	Hu Ping Zao	32.19 ± 2.07 B z	45.83 ± 0.79 C y	73.22 ± 0.34 A x	5.61
	Jun Zao	19.03 ± 1.46 C z	33.41 ± 1.34 D y	71.23 ± 1.43 A x	6.13
	*Z. acido-jujuba*	44.30 ± 2.54 A y	73.75 ± 0.27 A x	72.37 ± 0.77 A x	6.69
	Jurilovca	48.49 ± 0.99 A y	58.40 ± 1.13 B x	59.45 ± 1.64 B x	5.56
	*HSD_5%_*	*8.43*	*4.39*	*5.27*	
MeOH	Hu Ping Zao	38.26 ± 1.18 A y	25.95 ± 4.87 D z	69.57 ± 0.09 A x	12.56
	Jun Zao	8.97 ± 2.96 B y	51.55 ± 1.76 A x	45.65 ± 0.99 B x	8,99
	*Z. acido-jujuba*	27.03 ± 1.95 A y	33.90 ± 6.64 C y	70.46 ± 0.3 A x	17.34
	Jurilovca	30.67 ± 4.85 A z	43.35 ± 2.38 B y	65.59 ± 1.96 A x	14.4
	HSD_5%_	13.86	6.49	5.04	
Ae	Hu Ping Zao	11.6 ± 2.78 B y	38.43 ± 5.43 A x		16.93
	Jun Zao	3.51 ± 0.22 B y	40.05 ± 1.87 A x		5.23
	*Z. acido-jujuba*	29.96 ± 1.94 A y	52.7 ± 3.8 A x		11.84
	Jurilovca	23.55 ± 3.42 A x	35.27 ± 5.48 A x		17.94
	HSD_5%_	10.91	19.91		

EtOH—ethanol extract; MeOH—methanol extract; Ae—aqueous extract. Data represent the mean ± SE. Different letters (x, y, z) in the same row indicate significant differences between plant organs at *p* < 0.05 according to the Tukey test. Different capital letters (A, B, C, and D) in the same column indicate significant differences between genotypes at *p* < 0.05 according to the Tukey test.

**Table 4:** Antioxidant capacity (μmol TE/g DW) of the leaves, pulp, and stones of jujube genotypes using different extraction solvents.

Extraction	Genotype	Plant Organ			HSD_5%_
Solvent		Leaf	Pulp	Stone	
EtOH	Hu Ping Zao	13.07 ± 0.84 A x	3.13 ± 0.05 C y	2.67 ± 0.01 A y	2.09
	Jun Zao	6.17 ± 0.47 C x	2.19 ± 0.09 D y	2.59 ± 0.05 A y	1.2
	*Z. acido-jujuba*	9.96 ± 0.57 B x	4.85 ± 0.02 A y	2.65 ± 0.03 A z	1.43
	Jurilovca	3.48 ± 0.07 D y	4.06 ± 0.08 B x	2.23 ± 0.06 B z	0.31
	HSD_5%_	2.52	0.3	0.2	
MeOH	Hu Ping Zao	12.17 ± 0.37 A x	1.65 ± 0.2 B y	2.52 ± 0.01 A y	1.05
	Jun Zao	3.24 ± 0.49 C x	3.37 ± 0.11 A x	1.68 ± 0.04 C y	1.27
	*Z. acido-jujuba*	7.85 ± 0.56 B x	2.35 ± 0.37 AB y	2.62 ± 0.01 A y	1.68
	Jurilovca	2.21 ± 0.35 C x	3.18 ± 0.17 A x	2.38 ± 0.07 B x	0.99
	HSD_5%_	2.93	1.06	0.13	
Ae	Hu Ping Zao	4.03 ± 0.39 B x	2.84 ± 0.29 B y		1.16
	Jun Zao	1.55 ± 0.09 C y	2.74 ± 0.13 B x		0.44
	*Z. acido-jujuba*	10.16 ± 0.66 A x	3.75 ± 0.27 A y		1.97
	Jurilovca	1.49 ± 0.22 C x	2.27 ± 0.35 B x		1.14
	HSD_5%_	1.81	0.89		

EtOH—ethanol extract; MeOH—methanol extract; Ae—aqueous extract. Data represent the mean ± SE. Different letters (x, y, and z) in the same row indicate significant differences between plant organs at *p* < 0.05 according to the Tukey test. Different capital letters (A, B, C, and D) in the same column indicate significant differences between genotypes at *p* < 0.05 according to the Tukey test.

**Table 5:** FRAP (mM Trolox/L) in the leaves, pulp, and stones of jujube genotypes using different extraction solvents.

Extraction	Genotype	Plant Organ			HSD_5%_
Solvent		Leaf	Pulp	Stone	
EtOH	Hu Ping Zao	17.98 ± 0.18 C x	16.81 ± 0.46 B y	16.64 ± 0.03 B y	0.43
	Jun Zao	18.35 ± 0.14 B x	16.45 ± 0.12 B z	16.98 ± 0.12 A y	0.15
	*Z. acido-jujuba*	20.10 ± 0.17 A x	17.69 ± 0.08 A y	17.02 ± 0.03 A z	0.13
	Jurilovca	19.78 ± 0.01 A x	17.59 ± 0.03 A y	17.00 ± 0.08 A z	0.09
	HSD_5%_	0.33	0.72	0.17	
MeOH	Hu Ping Zao	17.24 ± 0.12 C x	17.03 ± 0.15 A y	16.93 ± 0.17 B y	0.16
	Jun Zao	18.01 ± 0.06 B x	17.02 ± 0.06 A z	17.32 ± 0.02 A y	0.06
	*Z. acido-jujuba*	16.25 ± 0.08 D y	15.83 ± 0.43 B z	16.75 ± 0.01 C x	0.28
	Jurilovca	18.95 ± 0.09 A x	16.70 ± 0.01 A y	16.59 ± 0.06 C z	0.07
	HSD_5%_	0.22	0.51	0.2	
Ae	Hu Ping Zao	17.56 ± 0.03 A x	16.08 ± 0.02 A y		0.06
	Jun Zao	16.76 ± 0.01 C x	16.24 ± 0.05 A y		0.08
	*Z. acido-jujuba*	17.31 ± 0.05 B x	15.26 ± 0.19 B y		0.46
	Jurilovca	17.22 ± 0.16 B x	16.43 ± 0.08 A y		0.14
	HSD_5%_	0.19	0.66		

EtOH—ethanol extract; MeOH—methanol extract; Ae—aqueous extract. Data represent the mean ± SE. Different letters (x, y, and z) in the same row indicate significant differences between plant organs at *p* < 0.05 according to the Tukey test. Different capital letters (A, B, C, and D) in the same column indicate significant differences between genotypes at *p* < 0.05 according to the Tukey test.

## Data Availability

Data are included in the article and Supplementary Materials.

## Supplementary Materials

The following supporting information can be downloaded at: https://www.mdpi.com/article/10.3390/plants14010134/s1, Figure S1: Biplot of DPPH radical scavenging activity using three extraction solvents in different plant organs of jujube genotypes; Figure S2: Biplot of antioxidant capacity using three extraction solvents in different plant organs of jujube genotypes; Figure S3: Biplot of FRAP using three extraction solvents in different plant organs of jujube genotypes; Table S1: Variance components of multiple regressions between fruit fresh weight and other fruit traits in jujube genotypes; Table S2: Pearson correlations between fruit-, pulp-, and stone-related traits in jujube genotypes; Table S3: Spearman correlations between extraction solvents regarding the ranks of jujube genotypes for DPPH radical scavenging activity; Table S4: Spearman correlations between extraction solvents regarding the ranks of jujube genotypes for antioxidant capacity.
