# Supplementary material for: Assessment of Fruit Traits and Antioxidant Capacity in Wild and Cultivated Genotypes of Ziziphus sp
Source: Plants (Basel). 2025 Jan 5;14(1):134. doi: 10.3390/plants14010134 (PMC11723295; doi:10.3390/plants14010134)
Supplement: Supplementary file 1 [file plants-14-00134-s001.zip › plants-3352957-supplementary.pdf]

Table S1. Variance components of multiple regressions between fruit fresh weight and other fruit traits in jujube genotypes

Source of variation	SS	DF	MS	F test
Hu Ping Zao				
Regression	9.2712	5	1.8542	86.56**
Fruit length (x_1)	2.4802	1	2.4802	115.77**
Fruit width (x_2)	0.0323	1	0.0323	1.51
Fruit shape index (x_3)	1.3046	1	1.3046	60.90**
Pulp fresh weight (x_4)	5.3368	1	5.3368	249.12**
Stone fresh weight (x_5)	0.1174	1	0.1174	5.48
Residual	0.0857	4	0.0214	
Total	9.3569	9		
$y = 2.37 -0.098x_1 -0.002x_2 +0.35x_3 +0.898x_4+5.22x_5$				
R^2 =0.9908; R^2_a =0.9792; SEE = 0.147; DW=2.59				
Source of variation	SS	DF	MS	F test
Jun Zao				
Regression	8.9544	5	1.7909	54.54**
Fruit length (x_1)	1.7323	1	1.7323	52.75**
Fruit width (x_2)	2.4374	1	2.4374	74.23**
Fruit shape index (x_3)	0.2480	1	0.2480	7.55
Pulp fresh weight (x_4)	4.5163	1	4.5163	137.53**
Stone fresh weight (x_5)	0.0205	1	0.0205	0.62
Residual	0.1314	4	0.0328	
Total	9.0858	9		
$y = -2.3 -0.159x_1 +0.174x_2 +2.46x_3 +0.978x4 +1.6x_5$				
R^2 =0.9855; R^2_a =0.9663; SEE = 0.099; DW=2.08				
Source of variation	SS	DF	MS	F test
Z. acido-jujuba				
Regression	0.0565	5	0.0113	23.46**
Fruit length (x_1)	0.0447	1	0.0447	92.77**
Fruit width (x_2)	0.0053	1	0.0053	11.01*
Fruit shape index (x_3)	0.0018	1	0.0018	3.74
Pulp fresh weight (x_4)	0.0043	1	0.0043	8.97*
Stone fresh weight (x_5)	0.0004	1	0.0004	0.83
Residual	0.0019	4	0.0005	
Total	0.0584	9		
$y = 0.4 +0.026x_1 +0.002x_2 -0.25x_3 +1.013x_4 -0.418x_5$				
R^2 =0.967; R^2_a =0.9269; SEE = 0.022; DW=2.68				
Source of variation	SS	DF	MS	F test
Jurilovca				
Regression	0.3193	5	0.0639	287.03**
Fruit length (x_1)	0.0068	1	0.0068	30.59**
Fruit width (x_2)	0.0114	1	0.0114	51.30**
Fruit shape index (x_3)	0.0031	1	0.0031	14.11**
Pulp fresh weight (x_4)	0.2979	1	0.2979	1338.70**
Stone fresh weight (x_5)	0.0001	1	0.0001	0.45
Residual	0.0009	4	0.0002	
Total	0.3202	9	0.0639	
$y = 1.49 +0.087x_1 -0.1x_2 -1.331x_3 +1.141x_4 +0.137x_5$				
R^2 =0.9972; R^2_a =0.9945; SEE = 0.014; DW=1.79				

** Significant at p≤0.05; ** Significant at p≤0.01.*

Table S2. Pearson correlations between fruits, pulp and stone related traits in jujube genotypes

Genotype	Traits	FL	FW	FSI	PFW	SFW	FFW
Hu Ping Zao	FL	-	0.190	0.321	0.612	0.703*	0.515
	FW	*p=0.599*	-	-0.859**	0.246	0.200	0.156
	FSI	*p=0.367*	*p=0.002*	-	0.121	0.193	0.163
	PFW	*p=0.060*	*p=0.493*	*p=0.739*	-	0.892***	0.980***
	SFW	*p=0.023*	*p=0.580*	*p=0.593*	*p<0.001*	-	0.900***
	FFW	*p=0.128*	*p=0.668*	*p=0.654*	*p<0.001*	*p<0.001*	-
Jun Zao	FL	-	0.493	0.055	0.506	0.422	0.437
	FW	*p=0.148*	-	-0.839**	0.672*	0.391	0.666*
	FSI	*p=0.880*	*p=0.002*	-	-0.445	-0.166	-0.478
	PFW	*p=0.136*	*p=0.033*	*p=0.198*	-	0.684*	0.988***
	SFW	*p=0.224*	*p=0.264*	*p=0.646*	*p=0.029*	-	0.705*
	FFW	*p=0.207*	*p=0.036*	*p=0.163*	*p <0.001*	*p=0.023*	-
Z. acido-jujuba	FL	-	0.875***	-0.207	0.824**	0.705*	0.875***
	FW	*p<0.001*	-	-0.653*	0.825**	0.640*	0.911***
	FSI	*p=0.566*	*p=0.041*	-	-0.355	-0.210	-0.459
	PFW	*p=0.003*	*p=0.003*	*p=0.314*	-	0.484	0.955***
	SFW	*p=0.023*	*p=0.046*	*p=0.561*	*p=0.156*	-	0.510
	FFW	*p<0.001*	*p<0.001*	*p=0.182*	*p <0.001*	*p=0.133*	-
Jurilovca	FL	-	0.806**	-0.304	-0.206	0.094	-0.146
	FW	*p=0.005*	-	-0.318	-0.253	-0.067	-0.229
	FSI	*p=0.392*	*p=0.371*	-	0.086	0.252	0.141
	PFW	*p=0.569*	*p=0.481*	*p=0.812*	-	0.739*	0.995***
	SFW	*p=0.796*	*p=0.854*	*p=0.483*	*p=0.015*	-	0.776**
	FFW	*p=0.688*	*p=0.524*	*p=0.697*	*p<0.001*	*p=0.008*	-

** Significant at p≤0.05; ** Significant at p≤0.01; ** *Significant at p≤0.001; n=10; FL-Fruit length; FW- Fruit width; FSI-Fruit shape index; PFW-Pulp fresh weight; SFW-Stone fresh weight; FFW-Fruit fresh weight*

Table S3. Sperman correlations between extraction solvents regarding the ranks of jujube genotypes for DPPH radical scavenging activity

Plant organ	Solvent	EtOH	MeOH	Ae
Leaf	EtOH	-	0.816**	0.943***
	MeOH	p=0.0012	-	0.577*
	Ae	p<0.0001	p=0.0495	-
Pulp	EtOH	-	-0.467	0.533
	MeOH	p=0.1258	-	-0.067
	Ae	p=0.0744	p=0.8361	-
Stone	EtOH	-	0.604*	
	MeOH	p=0.0375	-	

EtOH - Ethanol extract; MeOH- Methanol extract; Ae - Aqueous extract

** Significant at p≤0.05; ** Significant at p≤0.01; ** *Significant at p≤0.001; n=12.*

Table S4. Sperman correlations between extraction solvents regarding the ranks of jujube genotypes for antioxidant capacity

Plant organ	Solvent	EtOH	MeOH	Ae
Leaf	EtOH	-	0.949***	0.738**
	MeOH	p<0.0001	-	0.778**
	Ae	p=0.0061	p=0.0029	-
Pulp	EtOH	-	0.258	0.775**
	MeOH	p=0.4181	-	0.577*
	Ae	p=0.0031	p=0.0495	-
Stone	EtOH	-	0.816**	
	MeOH	p=0.0012	-	

EtOH - Ethanol extract; MeOH- Methanol extract; Ae - Aqueous extract

** Significant at p≤0.05; ** Significant at p≤0.01; ** *Significant at p≤0.001; n=12.*

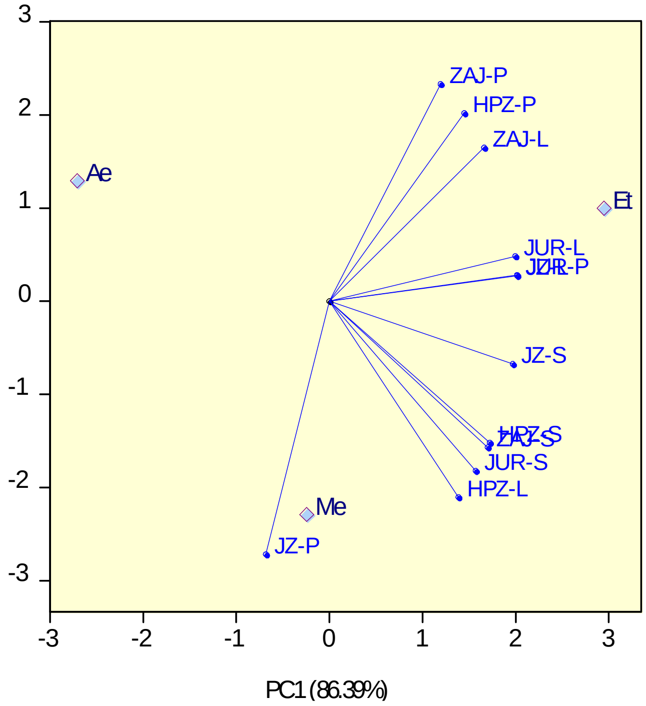

HPZ-L, Leaf of Hu Ping Zao; HPZ-P, Pulp of Hu Ping Zao; HPZ-S, Stone of Hu Ping Zao; JZ-L, Leaf of Jun Zao;JZ-P, Pulp of Jun Zao; JZ-S, Stone of Jun Zao; ZAJ-L, Leaf of Z. acido-jujuba; ZAJ-P, Pulp of Z. acido-jujuba;ZAJ-S, Stone of Z. acido-jujuba; JUR-L, Leaf of Jurilovca; JUR-P, Pulp of Jurilovca; JUR-S, Stone of Jurilovca;

Et - Ethanol extract; Me- Methanol extract; Ae - Aqueous extract

Figure S1. Biplot for DPPH radical scavenging activity using three extraction solvents in different plant organs of jujube genotypes

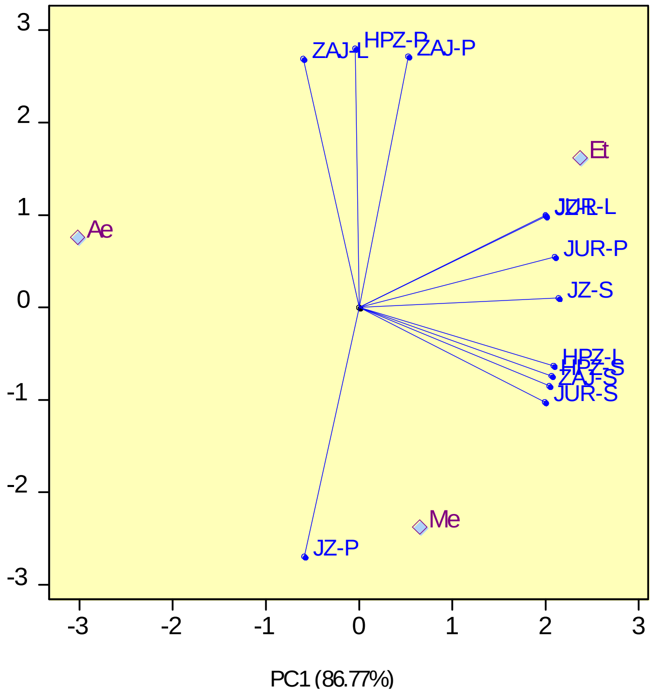

HPZ-L, Leaf of Hu Ping Zao; HPZ-P, Pulp of Hu Ping Zao; HPZ-S, Stone of Hu Ping Zao; JZ-L, Leaf of Jun Zao;JZ-P, Pulp of Jun Zao; JZ-S, Stone of Jun Zao; ZAJ-L, Leaf of Z. acido-jujuba; ZAJ-P, Pulp of Z. acido-jujuba; ZAJ-S, Stone of Z. acido-jujuba; JUR-L, Leaf of Jurilovca; JUR-P, Pulp of Jurilovca; JUR-S, Stone of Jurilovca

Et - Ethanol extract; Me- Methanol extract; Ae - Aqueous extract

Figure S2. Biplot for antioxidant capacity using three extraction solvents in different plant organs of jujube genotypes

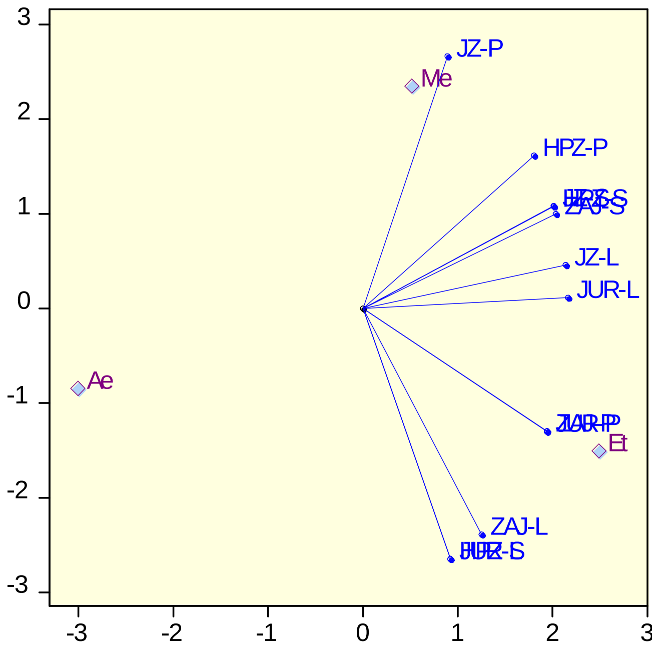

HPZ-L, Leaf of Hu Ping Zao; HPZ-P, Pulp of Hu Ping Zao; HPZ-S, Stone of Hu Ping Zao; JZ-L, Leaf of Jun Zao;JZ-P, Pulp of Jun Zao; JZ-S, Stone of Jun Zao; ZAJ-L, Leaf of Z. acido-jujuba; ZAJ-P, Pulp of Z. acido-jujuba; ZAJ-S, Stone of Z. acido-jujuba; JUR-L, Leaf of Jurilovca; JUR-P, Pulp of Jurilovca; JUR-S, Stone of Jurilovca

Et - Ethanol extract; Me- Methanol extract; Ae - Aqueous extract

Figure S3. Biplot for FRAP using three extraction solvents in different plant organs of jujube genotypes
